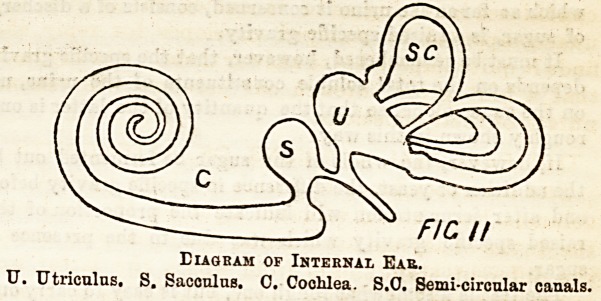# The Hospital Nursing Supplement

**Published:** 1895-11-16

**Authors:** 


### "The Hospital" Nursing Mirror.

BEING THE EXTRA NURSING SUPPLEMENT OF "THE HOSPITAL" NEWSPAPER.

[Contributions for this Supplement should be addressed to the Editor, THE HOSPITAL, 428, Strand, London, W.C., and should have the word "Nursing" plainly written in left-hand top corner of the envelope.]

### News from the Nursing World.

### OUR PRINCESS.

THE idea of a wedding gift for our Princess's youngest daughter, from Her Royal Highness's own Pension Fund nurses, has won universal approval. Every policy holder will doubtless wish to contribute the shilling suggested in our columns of last week, and all are invited to express an opinion as to the form which the gift should take. The subscriptions (with the senders' names and policy numbers) will be acknowledged in due course in THE HOSPITAL. The list will remain open until December 4th. Additional particulars may be found in last week's issue.

### NOTICE TO POLICY HOLDERS.

A BOOK now lies at THE HOSPITAL office to receive the signatures of the policy holders of the Royal National Pension Fund, to an address, or letter of thanks, to the donors of the handsome gift of £23,500. Nurses who are prevented by work or distance from calling at 428, Strand, can have their names inserted for them if they will write and request it, giving their policy number, full name and address, without delay, to the Editor of THE HOSPITAL. The list of signatures will be closed on November 25th.

### OUR NEEDLEWORK COMPETITION.

WE shall be glad to have all contributions connected with our annual needlework competition sent in on December 16th or 17th. Each parcel should be addressed "Needlework Competition," care of Editor of THE HOSPITAL, 428, Strand, London, and should contain name and permanent address of the sender. The prizes offered are: One guinea for the best flannel dressing-gown, 10s. for best bed jacket (for man or woman), 10s. for best flannel shirt, 5s. for best flannel petticoat, 5s. for best over-petticoat, 5s. for best knitted socks, 2s. 6d. for second best pair, 2s. 6d. for best warm vest (for man or woman). Other kinds of garments can also be sent to us for distribution, and should be marked "Not for competition."

### CERTIFICATES OF FIRST AID.

THE first aid certificates granted by the St. John Ambulance Association were presented to members of the Metropolitan Fire Brigade, Southwark Bridge Road, by Princess Christian, last week. There was a very interesting display of engines, which were galloped past the station, and then examples were given of the methods of saving life in fires. The proceedings, which were admirably arranged, attracted much local interest.

### NURSING AT THE SAMARITAN HOSPITAL.

THE nursing arrangements at the Samaritan Hospital for Women in Marylebone Road are not as advanced as might be expected from the outward appearance of this handsome modern building, for there is no nurses' home. The wards are well kept and orderly, but contain more superfluous furniture than is usually seen in such institutions. Doubtless this gives them a homelike look to patients accustomed to somewhat crowded apartments. The nurses employed in the larger wards sleep in another part of the hospital, but those on special duty have beds in the small wards with their patients, and, for the first day or two after operations the nurses do not even go downstairs to their meals. The little wards are made pretty and attractive by the nurse, who surrounds herself with her books, pictures, and other personal treasures, but we trust it will not be long ere the authorities of the Samaritan Hospital realise that it is neither necessary nor desirable to keep any woman on duty night and day even for a limited period and in comfortable quarters.

### EAST-END MOTHERS' HOME.

CLOTHES, books, nourishment, and flowers are required by the charity known as the "East-End Mothers' Home," and they will all be gratefully acknowledged by the Lady Superintendent, 396, Commercial Road, E. Necessary repairs to dilapidated premises have called for an outlay of £600, and the committee earnestly appeal for subscriptions to cover this amount. Midwifery and monthly nurses are trained at the Home, to which are admitted patients who cannot be otherwise properly nursed, and visiting nurses are provided for many poor women who desire not to be separated from their families whilst they are laid up. The structural improvements, which were absolutely necessary, have been economically carried out, and were inspected with interest on the 12th inst., when the Lord Mayor opened the new building in the presence of a party of ladies and gentlemen. The appearance of the in-patients testified to Miss Blomfield's care and good nursing, and the babies were particularly healthy-looking. Thirty-five pounds were collected at the meeting, and it is to be hoped that further help will be shortly forthcoming. The kindly rule which permits of the husbands spending an hour each evening at the bedsides of their wives is much appreciated. The men, in spite of their poverty, arrive clean and brushed up, and they are always eager to know if there are no coals to be carried up, or nurses' boxes to be moved, or any other small pieces of work, which they consider it a privilege to do for those who give such good care to their wives.

### TRAINING OF MALE NURSES.

AN Englishman who desires to get hospital training in medical and surgical nursing finds it impossible to do so in his own country, for no general infirmary or hospital has as yet consented to admit male probationers to its male wards, although we understand that the Seamen's Hospital, Greenwich, has the matter under serious consideration. Yet many gentlemen would gladly avail themselves of the services of a male nurse if they could be sure of obtaining a fully-trained and trustworthy one. The number of men desirous of undergoing the requisite preparation is too small to justify a fear of the female nurse being crowded out of any branch of the profession. The perfect condition of the wards at the fine French military hospital Val de Grâce at Paris shows what can be done by men who work under some supervision from the Sisters. The admirable neatness and order characteristic of this hospital contrasts favourably with the appearance of any other French hospitals. The male wards of many of our own large infirmaries, now often deplorably understaffed, might be utilised as training schools to the advantage of both male nurses and pauper patients.

### PREPAID REPLIES.

We are told by the matron of a nursing institution in the north that she gets a great many letters from candidates inquiring as to vacancies, but as these are unaccompanied by stamped envelopes for reply, she finds it quite impossible to answer them. She suggests that a hint given through our columns will remind nurses of their omissions.

### THE PERSONAL OPINIONS OF ATTENDANTS.

The correspondence to which we have opened our columns has thrown valuable light on the food and the duties of asylum attendants, and it has also shown the different standpoints from which the latter are apt to be regarded. It is impossible for us to insert all the letters addressed to us on the subject, but we are glad to give them due consideration, and we print as many as our limited space permits. We must again remind our friends that short, well-written letters are much more acceptable than those in which a superfluity of words tends to obscurity. In the recent epistles received from asylums it is observable that the grievances are generally of old-standing—in some cases the writers speaking of having put up with evils for a dozen years or more; and it seems as if our correspondents must have found some compensations for their trials, or they would hardly have endured them for such periods. There is apparent unanimity of opinion regarding the bad cooking for asylum workers, and this ought undoubtedly to be remedied. There must also be some explanation of another assertion made by several writers who speak of the absolute necessity for "religion" being left outside asylums! Surely their religious faith is too essentially a part of everyday life to be shed, like an outer garment, in any entrance hall. The dawn of better days is seen in the higher education and fuller training demanded in asylum workers, who will probably ere long be literally, as well as nominally, the *nurses* of the mentally afflicted. Now that evils have been so freely ventilated, it would be well for asylum workers to give equal publicity to ideas for their redress.

### TRAINING AT GLASGOW ROYAL INFIRMARY.

It is the custom at Glasgow Royal Infirmary for three months to be devoted to preliminary education in nursing by the candidates going in for the three years' course of training. Before admittance they have to satisfy the managers as to their general education, and the physician as to their health. The first course is on anatomy, lectures being given twice a week by Professor Clark; the second is on physiology, by Professor Barlow; and the third on hygiene, by Professor Glaister. Candidates who succeed in passing the examination held at the conclusion of the first course are eligible for the second, which consists of clinical lectures on surgical cases by Dr. Adams, and on medical cases by Dr. Wallace Anderson. Instruction in ward work and cookery is given by Mrs. Strong (the matron), aided by the housekeeper, and it comprises the cleaning of ward appliances, the application of surgical dressings, and the care of instruments. The cookery course consists of four demonstrations and six practical lessons.

### ON AND OFF DUTY.

"I haven't seen Nurse Smith to-day, matron, and Probationer Jones is not in her ward either," remarked a well-meaning member of the House Committee, who looked in "just to see how things were going on at the hospital." "They are both off duty; I have given them extra time to-day, as the committee wished." "But what about the patients, matron?" With a slight smile, she answered, "I am looking after the ward myself till Sister comes back at six." "But surely you have other things to do?" "Yes; they will get done by and by. Of course, it means extra time on duty for some people, to secure extra time off for others, but I don't mind it for myself." The member of committee glanced at the well-kept ward and the carefully-tended patients. "I'm sorry to miss the nurses," he said; "I like to see them all on duty." But as he went home he felt some misgiving about the request he had scornfully rejected at a recent meeting. The matron had asked for an extra nurse to enable her to give increased leave of absence to her staff, but she was told that she must manage without adding to working expenses. "I suppose she and Sister will knock up now," grumbled the committee man, "and that will cost more money in the end."

### UNQUALIFIED TO ADVISE.

There seems to be an impression that anyone who has visited a hospital half-a-dozen times, or has the good fortune to possess as a friend a "paying pro.," must be competent to write upon the profession or art of nursing. Hence not infrequently articles appear in the ladies' lay press to instruct the would-be probationer what to do. These compositions would often be amusing if they were not irritatingly misleading. Girls are advised to select one of the principal hospitals whose rules appear likely to suit them, but they are not warned of the small chance of a vacancy awaiting them, nor of the personal fitness exacted in themselves, and in other particulars the information given is often evidently founded on fancy, not fact.

### SHORT ITEMS.

The third annual report of the Women's Convalescent Home Association shows satisfactory results, 365 persons having been admitted to "St. Olave's," West-Cliff-on-Sea, in the last twelve months. Applications should be addressed to Secretary, 29, Memorial Hall, E.C.—The Tavistock Board of Guardians agreed at a recent meeting, after some discussion, to continue the employment of pauper help in the sick wards.—The establishment of a trained district nurse at Enniskillen is under discussion, and also affiliation with the Queen's Jubilee Institute.—The Guardians decided to give a testimonial to a nurse at St. Olave's Infirmary who had broken her agreement, and they deliberately ignored the opinion of the medical superintendent in the matter.

### Elementary Physiology for Nurses.

By C. F. Marshall, late Surgical Registrar Hospital for Sick Children, Great Ormond Street.

XV.—THE SENSE ORGANS (*continued*).

*The Sense of Pressure.*—The forehead, temple, and back of the hand are the parts most sensitive to the pressure sense, and these parts can distinguish between weights in the ratio of 29 to 30.

*The Muscular Sense* is the consciousness of the amount of muscular exertion necessary to support a body, or to exert pressure on it. If we hold a weight in our hand when the hand is supported on a table, we are conscious only of the pressure exerted on the hand by the weight, or the sense of pressure. If, however, the hand is unsupported, we are also conscious of the force necessary to support the weight. This is the "muscular sense," as distinguished from the sense of pressure. It is this sense which guides all our muscular actions and all the movements of the body. The muscular sense is finer than the sense of pressure, and can distinguish between weights in a ratio of 39 to 40.

*The Sense of Temperature.*—This is relative rather than absolute, as is shown by the following experiment. If we take three basins of water, one hot, one cold, and the third lukewarm, and put one hand into the hot water and the other into the cold water, and then put both hands into the lukewarm water, the latter will feel cold to the one hand and hot to the other. There are supposed to be special nerve endings in the skin which are sensitive to the different degrees of temperature, apart from those which are sensitive to touch.

*The Sense of Taste.*—This is situated chiefly in the tongue, probably aided by the palate. This sense is very inaccurate. In order to taste a substance it must be in solution, and the tongue must be moist, for a piece of sugar on a dry tongue will not be tasted. Much of the sense of taste, as it is generally known, is really due to smell, and it is to this that the various flavours of wines are largely due. We all know how a cold in the nose destroys to a great extent the sense of taste. There are special nerve endings for taste situated in the papillæ covering the tongue and connected with two of the cranial nerves, viz., the trigeminal and the glosso-pharyngeal.

*The Sense of Smell.*—In order to smell a substance it must be in a gaseous or vaporous condition. The sense of smell is conveyed to the brain by the olfactory nerve, which is distributed to the upper part of the mucous membrane inside the nose. The sense of smell is not well developed in man, but in some animals, such as deer, and in some dogs, it is very acute.

*The Sense of Hearing.*—This is far more definite and of greater interest than the senses considered alone. It is this

FIG 10

Diagram of Simplest Form of Ear.

A. Auditory nerve sending branches to the columnar cells (C). H. Hair-like processes of auditory cells.

sense that enables sounds to be appreciated by the brain. Sound is caused by vibrations communicated by the air or by a solid body. There must be some medium by which the vibrations are communicated, for sound cannot travel in a vacuum.

*The Ear.*—The simplest form of ear, such as is found in jelly fish, snails, &c., is a little closed sac imbedded in the body and having at one part columnar epithelial cells, which are furnished with hair-like processes, and to which the ends of the nerve of hearing are connected. The sac is filled with fluid.

In the higher animals and in man the ear is essentially similar, but far more complicated.

*The Ear in Man.*—The essential part is the internal ear imbedded in the skull. This in its early stages of development is a simple sac like the ear of the snail, but during development it becomes complicated and divided into several parts. The sac is filled with fluid called endolymph; surrounding it in its bony cavity in the skull is more fluid, the

FIG 11

Diagram of Internal Ear.

U. Utriculus. S. Sacculus. C. Cochlea. S.C. Semi-circular canals.

perilymph. The main part of the internal ear or vestibule is divided into two sacs—a smaller one, the sacculus, and a larger one, the utriculus. From the sacculus arises the cochlea, a spiral with two and a half turns. From the utriculus arise three semi-circular canals, which are situated in three planes at right angles to each other. The ampullæ are dilations at the ends of the semi-circular canals where they join the utriculus. Special nerve endings are situated in the sacculus, utriculus, cochlea, and ampullæ.

### Minor Appointments.

Cottage Hospital, Hatfield Broad Oak.—Miss Emma M. Dawe has been appointed sister-in-charge of the Cottage Hospital, Hatfield Broad Oak, Harlow,' Essex. We congratulate her on her appointment.

Indian Nursing Service.—Miss Ellen Barker, who was trained at the Nightingale School, St. Thomas's Hospital, has also been made a Sister in the Indian Nursing Service. Miss Barker has been doing private nursing, as a member of the Nurses' Co-operation, 8, New Cavendish Street, and we wish continued success to the nurse whose personal courage was mentioned in The Hospital of October 19th.

Indian Nursing Service.—Miss Helen Adelaide Mills has received an appointment as Sister in the Indian Nursing Service. She was trained in the Nightingale School at St. Thomas's Hospital, and was afterwards Sister of Christian Ward. Miss Mills' departure is viewed with regret by many friends and all her fellow-workers, with whom she has been most popular, and she takes with her very cordial good wishes and congratulations.

### "Nursing Notes" Exhibition.

We regret that by an oversight an "aseptic locker," shown at the above exhibition, and of which a sketch appeared in The Hospital for November 9th, was in the description attributed to Messrs. Down Brothers, instead of to the Evelina Hospital for Children, from whence it really came, the makers being Messrs. Ponder and Baker.

### On Urine Testing.

III.—SUGAR AND UREA.

The specific gravity of the urine being the measure of the amount of material which is dissolved in it, it is obvious that the persistence, amid all conditions, of a very low specific gravity is an indication of degeneration of the kidney, of its failure as an excretory organ, for the efficacy of the kidney is shown not by the amount of water which exudes, but by the quantity of waste products which that water carries off. On the other hand, when the amount of work being performed by the body is not in excess, an excessive specific gravity (taking an average sample from the whole twenty-four hours' secretion), especially when the quantity of urine is also large, shows that the kidneys are discharging something abnormal or excessive.

It thus happens that the first rough proof of diabetes, which so far as the urine is concerned, consists of a discharge of sugar, is a raised specific gravity.

It must be remembered, however, that the specific gravity depends on the total soluble constituents of the urine, not on the sugar alone, so that the quantity of the latter is only roughly shown in this way.

If, however, the whole of the sugar be fermented out by the addition of yeast, the difference in specific gravity before and after fermentation will indicate the proportion of this raised specific gravity which was due to the presence of sugar.

The test is a somewhat rough one, but is easy to carry out. A small lump of German yeast (the size of a walnut) is added to four ounces of urine, the specific gravity of which has first been carefully taken and noted; this urine is placed in a wide-mouthed bottle (12 oz.), a pickle bottle for example corked, but with an opening cut in the cork to allow the carbonic acid to escape. It is then kept in a moderately warm situation, and, in the course of twenty-four hours, or when the fermentation is completed, it is allowed to cool, and its specific gravity is taken again at the same temperature as before. The object of the large bottle is to leave room for froth. Perhaps a simpler way is to take two bottles, each containing the same quantity of urine, but one containing the yeast and the other not. Both bottles being kept in the same condition and at the same temperature, the amount of sugar is indicated by the difference in the specific gravities of the two at the end of the experiment. "It has been found empirically that each degree of specific gravity lost by fermentation corresponds with one grain of sugar per fluid ounce of urine" (Finlayson). The ordinary method for testing for the presence of sugar is by the use of Fehling's solution, or one of its substitutes. A test tube is filled about one inch deep with the solution, which is of a dark blue colour; this is then boiled. If it remain unchanged in colour it may be considered to be in good condition and fit for use (this precaution is necessary because it tends to spoil by keeping). The urine is then slowly added, drop by drop, when, if diabetic sugar be present in any quantity, the colour changes and a yellowish or reddish precipitate occurs.

If this does not take place at once more urine may be added (but the total amount of urine used must not exceed the original amount of the test), and the mixture again boiled and allowed to cool, when if no yellow or red precipitate falls it may be pronounced free from sugar.

To estimate the quantity of sugar by Fehling's test requires special apparatus, and is hardly likely to fall within a nurse's duty.

The cases in which the use of Fehling's test is most likely to be required are those in which diabetes has been practically recovered from, but tends to return whenever dietary rules are too far relaxed; the fermentation test, on the contrary is most commonly required in the course of the disease for the purpose of telling, as nearly as may be, the quantity of sugar p ssed per day.

It is obvious that in any case the total urine passed in the twenty-four hours must be carefully measured, and that the specimen examined must be a sample of the whole. The amount of sugar per ounce multiplied by the number of ounces will then give the total weight passed in the twenty-four hours. It is, however, very common for a doctor in a known case not to ask for more particulars than the total quantity and the average specific gravity. If these are regularly tabulated a very fair conjecture can be made of the progress of the disease.

The estimation of urea need not here be entered into. It is usually done by measuring the amount of gas evolved from a mixture of a measured quantity of urine with certain solutions. Various forms, both of apparatus and of solution, are in use for the purpose, and the directions given with each must be strictly adhered to. With almost any of them the results are sufficiently accurate for clinical purposes; the great thing is to follow carefully the instructions given with each particular form of apparatus.

### London Nurses' Training Schools.

LECTURES TO PROBATIONERS.

### QUEEN VICTORIA'S JUBILEE INSTITUTE FOR NURSES.

Central Training Home, 23, Bloomsbury Square.—Winter Course of Lectures.

Mrs Dowson, M.D., "Hygiene," commenced first Friday in September; 13 lectures and examination; Tuesdays and Fridays, three p.m.

Dr. Coupland, "Fevers," commenced fourth Monday in October; 13 lectures and examination; Mondays and Wednesdays, half-past three p.m.

Mrs. Scharlieb, M.D., "Obstetrics," commence second Tuesday in December; 13 lectures and examination; Tuesdays and Fridays, three p.m.

### ROYAL FREE HOSPITAL, GRAY'S INN ROAD, W.C.

Lectures are given to the nurses at this hospital by Willmott Evans, M.D., F.R.C.S., Registrar to the hospital. He gives twelve lectures (which began on September 30th) before Christmas. The probationers are examined by a senior physician and senior surgeon on the visiting staff, each of whom sets a paper, and there is also a *viva voce* examination. The lectures before Christmas are on "Anatomy and Physiology."

### ST. GEORGE'S HOSPITAL.

A course of lectures to the nursing staff will be given on Tuesdays and Wednesdays, at three p.m., by Dr. Penrose and Mr. Dent. Dr. Penrose will lecture on Wednesdays, Mr. Dent on Tuesdays.

### KING'S COLLEGE HOSPITAL.

Lectures by Dr. John Curnow, Dean of the Medical School, on Fridays at 6 p.m.; November 15th and 22nd, "The Lower Extremity"; November 29th and December 6th, "The Abdomen"; December 13th, "The Throat"; December 20th, "The Neck."

The above list is the first part of the elementary course; every other year an advanced course is given. Classes by the Home Sister on Wednesdays at 10 a.m. and 8 p.m.

*** Particulars are not yet forthcoming from University College and Westminster Hospitals.

### Wants and Workers.

[The attention of correspondents is directed to the fact that "Helps in Sickness and to Health" (Scientific Press, 428, Strand) will enable them promptly to find the most suitable accommodation for difficult or special cases.]

May I trouble some reader of The Hospital to give me a few suggestions as to the outfit rquired for a missionary's young wife in Southern India? I should be glad to know kind of dresses, underclothing, colour of stockings recommended—*India.*

### Nursing in Florence.

I.—OSPEDALE DI SANTA MARIA NUOVA.

This, the largest and oldest, is situated in the Piazza of the same name. It was founded by Folco Portinari, the father of Dante's Beatrice. Noticing that his daughter occupied herself greatly in nursing the aged women of the city, he thought to please her and do a good work by building a hospital where the sick might be cared for.

It is an enormous place, containing sixteen corsie or wards for men and eighteen for women, with about eight hundred beds and a bewildering number of passages, courts, and gardens. It is in the charge of three physicians, three surgeons, and seven medical and surgical assistants.

The nursing is done by a few lay helpers taken from any class of society, and still fewer Sisters of Charity, while on the men's side it is done by laymen under a *corporale*. The nursing staff is quite unequal to the demands made upon it; for example, one Sister said she had the sole care of 172 beds.

Admission is obtained by certificate from a parish doctor or by a syndic's "fede de miserabilita," except, of course, in cases of street accidents or suicides, which are brought in without ceremony by members of the Misericordia. The fee per day for each is from two francs upwards.

We fell into the ranks of the ordinary daily visitors, who are admitted five times in the week from twelve to one, thinking we should see more of the life in the hospital by so doing. To our surprise the visitors were allowed to take in food and drink to the patients: bread, wine, meat, fowls, broth, milk —anything, indeed, except cake, pork, fruit, and vegetables, which are strictly forbidden. We followed on into the wards and saw all these eatables given to the sick without let or hindrance, and as there was neither cupboard nor table by the bedside, the patients put all that was given them under their pillows and under their backs, where also they kept their soiled linen, in readiness for their friends to take home to wash. Being visiting day the babel of tongues, the tramping to and fro, and the utter disregard of dying patients added to the sense of depression, discomfort, and confusion. On the men's side are four wards in the shape of a Greek cross, immensely lofty, and lighted by large windows near the ceiling, which in winter cannot be opened sufficiently for ventilation owing to there being no means of properly heating the wards. The first we entered was a monster deposit ward or corsia, which opens upon the yard, where all male cases of whatever kind are taken on arrival. If the injuries can be cured in a few hours or days they are left here, otherwise, after the doctors have seen them, they are drafted off to other wards, medical or surgical. Half of this ward was bricked up, but still it was as big as a church, and the few dozen beds in it were almost lost sight of in its huge proportions.

The deposit ward on the women's side was also a huge place, cold and cheerless. There is no separate ward for children, who are placed with grown-up people. We went through a succession of wards on both sides; the floors were all of bare red tiles, and must strike very cold to the bare feet, as there is neither carpet nor wadded shoes on which to stand. In order to keep them clean and free of dust, the floors are sprinkled with some diluted acid, which gives off an unpleasant smell.

The wards all looked cold and bare after ours; even as the nurses, in no uniform and with untidy hair, seemed slovenly after our trim, neat attendants at home. The iron beds, spring mattresses, and coarse linen sheets were all good, and the covering sufficient. Beside the beds the only articles in the ward were two chairs and a table. Between each bed there is about one yard of space; here a tiny shelf is fixed into the wall just large enough to hold the medicine, water-bottle, and dirty glass. Several of the chamber utensils were running over by the beds, and the sanitary arrangements will not bear speaking of.

The patients complained greatly of their food, which we did not think bad, only that it came to them nearly cold and very unappetising to sick people. The coffee was excellent.

Our opinion is that the nursing and supervision are utterly inadequate, and that the state of things within it would not be tolerated for a moment elsewhere. The night attendants are so tired that they sleep soundly at their post.

There is a magnificent library in this hospital for doctors and students, but none for the patients. One of the large wards is boarded off, to be used in case of epidemic, typhoid, or infectious disease. The nurses get about £20 a year, with food, and an increase of one-tenth every five years.

### Our American Letter.

(Contributed.)

An appointment recently made has given great satisfaction in the nursing world, for Mrs. Quintard has been named directress of nurses at the new St. Luke's Hospital in New York City. Mrs. Quintard received her training at the New Haven Hospital, Connecticut, where she was afterwards superintendent of nursing; and since taking up the duties of that office in 1890 she has displayed great administrative abilities.

Another important appointment is that of Miss Linda Richards to Hartford Hospital. The vacancy left by her leaving the Brooklyn Homœopathic Hospital, where she has done excellent work, has been filled up by Miss Anna Alline.

Miss Delano's resignation of the post of instructor of nurses at the hospital of the University of Pennsylvania has led to the promotion of Miss Bernard to be her successor in office. Miss Bernard was trained at the Johns Hopkins Hospital.

An American pension fund for nurses which has been organised at Philadelphia aspires to provide in the future old-age pensions and sick pay for disabled nurses in the United States. It is proposed to raise the necessary funds for the purpose by means of fancy sales and bazaars. The first of these will take place in the Natatorium, Philadelphia, December 2nd to 7th.

An arrangement has been made at the Women's Hospital, New York, which should prove acceptable to graduates desiring to add special experience in gynæcology to their previous training. Only those who have obtained diplomas from a nurse-training school are eligible for admission to the course, which will cover nine months. Miss Frances Flower, who is superintendent of the Woman's Hospital, will give further particulars to applicants.

Fifteen nurses received diplomas last month at the New York Hospital. A very pleasant gathering of friends took place on the occasion.

At the Baltimore University the first graduates to receive a two years' course of training were recently presented with diplomas.

The next conference of the American superintendents is fixed for February, when the question of a uniform curriculum and other matters will receive due attention.

### Where to Go.

Holborn Town Hall.—The annual ball in aid of the Royal Free Hospital, Gray's Inn Road, is fixed for December 5th.

Winter Sale of the Working Ladies' Guild will be held at 24, Park Lane, W., November 27th, 28th, and 29th, 1895, from twelve to six each day. H.R.H. Princess Beatrice will open the sale on the 27th.

Royal British Nurses' Association, 17, Old Cavendish Street.—Lecture by Dr. Louis Parkes at eight p.m. on November 22nd. Subject, "The Importance of Breathing Fresh Air."

### Everybody's Opinion.

[Correspondence on all subjects is invited, but we cannot in any way be responsible for the opinions expressed by our correspondents. No communications can be entertained if the name and address of the correspondent is not given, or unless one side of the paper only be written on.]

### NURSING AT THE RADCLIFFE INFIRMARY.

"Medicus" writes: Your paper has more than once criticised our infirmary here—the Radcliffe. There is one point, however, that has not been touched on which I should be glad to call to the notice of your readers. There is a cruel rule in force as regards the nurses that certainly ought to be repealed. The nurses are on duty from seven a.m. to half-past eight p.m. They are in the wards at least ten hours, and during all this time they are forbidden to sit down. What excuse is there for such a regulation as this? In hospitals under more humane management this is not the case, but the nurses are advised to sit down as much as they can. There is another thing that ought to be altered. Surely the hours named above are long enough for duty, but if any nurses are ill the others have to take their place and do extra work, instead of outside nurses being engaged, as would be easy enough. Is not this sweating pure and simple?

[We have communicated with the infirmary authorities, and are assured that there is no rule, written or otherwise, in regard to nurses sitting down in the wards, and that in fact they do sit down, and have more opportunity of doing so than in many other institutions. The authorities urge that a reference to our article in The Hospital of November 2nd on the proportion of nurses to patients in London and provincial hospitals shows that the staff at the Radcliffe Infirmary is greatly above the average. As regards outside nurses, we are informed that they are employed when necessary, and at the present time four of them are at work in the infirmary.—Ed., *T. H.*]

### ASYLUM WORKERS.

"An Asylum Hospital Nurse" writes: Permit me to enter a protest against the letter of "An Old Nurse" which appeared in The Hospital. I changed hospital for asylum work some months ago; and, while acknowledging that hospital work is more interesting and more comfortable than asylum work, I can still recognise how much good there is to do in an asylum. The doctors invariably, and my patients generally, have treated me with quite as much respect as I ever received in hospital; and I have never had a blow from any of my patients. In the hospital where I was trained, asylum nurses were made welcome for short training in the general wards, and I do not believe that any intelligent matron refused a candidate simply because she is an asylum nurse. A woman with principle and self-respect, instead of becoming degraded by the moral tone of those around her, may do much to raising it to a higher level. It is absolutely incorrect to say that religion must be left outside asylum walls. I find it quite possible to conduct prayers in my ward every evening, and my patients usually listen with respectful attention, and though not previously accustomed to the Church of England service, nor yet under any obligation to attend it, I find worship as possible here as elsewhere.

### THE LIVES OF ASYLUM WORKERS.

"Another Asylum Nurse" writes: In a copy of The Hospital dated October 26th I read with much interest "An Old Nurse's" letter anent "The Lives of Asylum Workers." Her experience is, in a great measure, the experience of the generality of asylum nurses. There are evils to be put up with that can never be altered, owing to the nature of the people who are there from necessity, not choice. But for one of the most serious evils at present existing the patients are in no way accountable, and it could be removed if interest were rightly aroused. I refer to the badly-cooked food. There is a saying that "God sends the meat and the devil sends the cooks." Certainly culinary productions destined for the nurses' consumption verify the truth of this saying. Anything is good enough for the nurses, and the idea of paying any attention to their food after it is put in the oven seems considered preposterous. Far more attention is paid to the cooking of the patients' food, because the medical superintendent may walk into the dining hall at any moment, and be deafened with clamouring complaints from the more sensible inmates. But the nurses' complaints are seldom heard, and their food, after it is cooked, is seldom seen by the superintendent. He would consider it an intrusion to walk into the nurses' mess-room and scrutinize their dinners. The medical staff in asylums are most considerate and kind in their treatment of nurses, and willing to redress any grievance brought under their notice. But many nurses would rather spend half their wages in procuring food for themselves than make complaints to a busy superintendent. Most asylum nurses are more or less martyrs to indigestion owing to ill-cooked food, and it would benefit all concerned if more attention was paid to their diet, and there would be fewer names on the sick list. As to the doctors "sleeping peacefully in a private wing, and being provided with a three course dinner," what nurse would begrudge them either? No reasonable nurse expects to be housed and fed like one of the medical staff, but it is not right that she should be victimised by a grudging cook's meanness, when a little supervision from the right quarter would work wonders. "An Olp Nurse" says we are "reckoned by outsiders as a lazy class, and not of the strictest character." A grievance, no doubt, but surely a petty one, if a nurse has a clear conscience. Seven years' experience has shown me the advantages as well as the drawbacks of asylums, for surely it is an advantage to have a good library, to attend lectures on mental diseases and mental nursing, to practise singing in the choir, to have the use of a piano, to take part in dramatic entertainments, to have a half-day off duty every week and a whole Sunday every three weeks, to have twelve days' annual leave, and last, but not least, the option of associating with one's fellow workers, or isolating oneself when duty hours are over, which a room of one's own renders possible.

### REAL AND SHAM NURSES.

"A Provincial Trained Nurse" writes: After reading your correspondents' letters of last week I am now more than ever impressed with the urgent need and desirability of a strenuous effort being made to check the abuse of our uniform. I marvel that the heads of the many institutions formed for the benefit of nurses, as well as the matrons of our training schools, do not take this matter up. I suggest that a voluntary syndicate be formed by nurses or anyone interested in nurses, and a petition drawn up for presentation to H.R.H. the Princess of Wales—"our Princess"—praying for her intervention in this matter. The said petition could lie at a chosen place for the signatures of nurses in London, and for the entry of the names of those who are unable to personally attest their signature but signify their approval by letter or postcard. A notice in this paper would certainly be sufficient to bring it under the notice of most, if not all, nurses, and I should think at the same time gain their willing and hearty co-operation. I leave it to older and more experienced heads than mine to suggest an improvement on the above, or propound a more effectual scheme. My time is limited, but what little leisure time I have I would willingly place at the disposal of anyone who would care to discuss the probable success of such a movement.

### The Princess Maud Marriage Present Fund.

Nurses sending contributions to this Fund are requested to write outside the envelope in the left-hand corner the words "Princess Maud," as this will save considerable trouble in dealing with the corrrespondence. Nurses are reminded that all letters on this subject should be addressed to the Manager of The Hospital, 428, Strand, London, W.C., and not to the office of the Pension Fund. It is disappointing to find that, despite the request last week, many nurses have addressed the secretary of the Pension Fund on this subject, and so added to the pressure at the Pension Fund offices which we invited nurses to combine to relieve as much as possible. The following subscriptions have been received at The Hospital Office this week:—

First List of Contributors.

| | s. | d. | | s. | d. |
|---|---|---|---|---|---|
| F. E. Manning ... ... | 1 | 0 | A. J. Bullock ... ... | 1 | 0 |
| M. A. Price ... ... | 1 | 0 | L. C. F. ... ... ... | 1 | 0 |
| M. Hawkins ... ... | 1 | 0 | S. Dutton ... ... ... | 1 | 0 |
| E Leedham ... ... | 1 | 0 | I. R. Maxwell ... ... | 1 | 0 |
| S. A. Silverwood ... | 1 | 0 | H. W. ... ... ... ... | 1 | 0 |
| R. Morris ... ... ... | 1 | 0 | R. W. ... ... ... ... | 1 | 0 |
| S. E. Garnham ... ... | 1 | 0 | F. M. Rabson ... ... | 1 | 0 |
| A. F. Poole ... ... | 1 | 0 | E. L. Barrow ... ... | 2 | 6 |
| H. I. Proschwitzky ... | 1 | 0 | E. Davey ... ... ... | 1 | 6 |
| E. Elliot ... ... ... | 1 | 0 | E. Custance ... ... | 1 | 0 |
| R. Maunder ... ... | 1 | 0 | C. A. Hoobs ... ... | 1 | 0 |
| J. Connell ... ... ... | 2 | 0 | E. Saintsbury ... ... | 1 | 0 |
| E. Nicholson ... ... | 2 | 0 | E. Barkwith ... ... | 2 | 0 |
| A. E. Stewart ... ... | 1 | 0 | M. E. Smith ... ... | 1 | 0 |
| E. R. Wallbank... ... | 1 | 0 | Nurse Talbot ... ... | 1 | 0 |
| G. A. Wyld ... ... | 1 | 0 | S. Anthony... ... ... | 1 | 0 |
| M. A. Reeve ... ... | 1 | 0 | B. J. S. Westbrook ... | 1 | 0 |
| M. Asser ... ... ... | 1 | 0 | E. Manning ... ... | 1 | 0 |
| S. J. Browne ... ... | 1 | 0 | H. Randall... ... ... | 1 | 0 |
| S. J. Weston ... ... | 5 | 0 | E. Peach ... ... ... | 1 | 0 |
| Anon ... ... ... ... | 1 | 0 | E. K. Holmes ... ... | 1 | 0 |
| L. Wilkinson ... ... | 2 | 0 | M. Coupland ... ... | 1 | 0 |
| J. Riddach... ... ... | 1 | 0 | M. Geard ... ... ... | 2 | 0 |
| E. M. A. Sherring ... | 1 | 0 | P. Legate ... ... ... | 1 | 0 |
| J. R. Dinwoodie ... | 1 | 0 | Nurse Grey... ... ... | 1 | 0 |
| G. Lees ... ... ... | 1 | 0 | S. E. Ransford ... ... | 1 | 0 |
| H. M. Walker ... ... | 2 | 0 | J. P. ... ... ... ... | 1 | 0 |
| M. A. Winning... ... | 1 | 0 | M. A. C. ... ... ... | 1 | 0 |
| A. Arnold ... ... ... | 1 | 0 | M. J. K. ... ... ... | 1 | 0 |
| E. Lander ... ... ... | 2 | 0 | S. C. Townshend ... | 1 | 0 |
| O. Ashley ... ... ... | 1 | 0 | J. A. Greene ... ... | 1 | 0 |
| M. A. B. Groom ... | 1 | 0 | | | |

### Notes and Queries.

### Queries.

(29) *Mental.*—Can you tell me anything of a guild started for the infirm in mind?—*Nurse Jane.*

(30) *Books.*—Please give me the names of technical books of use to nurses.—*L. M. N.*

(31) *Homes.*—Can you tell me of any homes in London or elsewhere in which would be admitted a child of nine, who cannot walk? He is an idiot and hydrocephalic, and his parents are very poor, father an invalid.—*Florentices.*

(32) *Oculists.*—Will you kindly give me the names of some leading oculists for a young man whose sight is getting very bad?—*Policy* 2,939.

(33) *Insane.*—Having heard that a convalescent home for insane persons is about to be opened I shall be grateful for a few particulars. I am interested in a young woman suffering from melancholia.—*M.*

(34) *Madras.*—Will you kindly tell me how I can get all the information about the Madras General Hospital?—*Teddie.*

(35) *Midwifery.*—Can you tell me of a centre in Lancashire where lectures on midwifery are given?—*Monthly Nurse.*

### Answers.

(29) *Mental* (*Nurse Jane*).—Perhaps you mean "Church Guild of Friends of the Infirm in Mind," of which you can get particulars from the Rev. Hy. Hawkins, chaplain, Colney Hatch, N.

(30) *Books* (*L. M. N.*).—There is a good list of nursing books in "How to Become a Nurse" (Scientific Press).

(31) *Homes* (*Florentices*).—See list of homes in "Burdett's Hospital Annual."

(32) *Oculists* (*Policy* 2,939).—We are very sorry not to answer your question, but we never give doctors' names. You can get the addresses of ophthalmic hospitals in London and elsewhere from "Burdett's Hospital Annual." It is published by the Scientific Press.

(34) *Madras* (*Teddie*).—By writing direct to the matron at the hospital.

(35) *Midwifery* (*Monthly Nurse*).—You can get the best information on the subject of your letter from the hon. secretary of the Midwives' Club, 12, Buckingham Street, Strand, London. In writing to her you should enclose a stamped envelope.

### Appointments.

[It is requested that successful candidates will send a copy of their applications and testimonials, with date of election, to The Editor, The Lodge, Porchester Square, W.]

Rainhill County Asylum.—Miss M. Burns has been appointed head nurse (matron) of the Richmond District Asylum, Dublin. She was trained at the Queen's Hospital, Birmingham, where she afterwards held the post of ward sister, and for the last three years has been head nurse (assistant matron) at the County Asylum, Rainhill. We wish Miss Burns every success in her new work.

Birkenhead Union Hospital and Infirmary.—Miss Francis E. Marquardt has been made Superintendent Nurse of this institution. She was trained at the Poplar Hospital for Accidents, and Royal Free Hospital, Gray's Inn Road; she was ward Sister at the latter hospital; head nurse and superintendent of nurses at Barbadoes General Hospital; and night superintendent nurse at Greenwich Infirmary. We congratulate Miss Marquardt on her appointment, and wish her continued success.

East London Nursing Society.—Miss R. Ashbee has been appointed matron of the central division of this society. She was trained in the Nightingale School at St. Thomas's Hospital, was a sister at Guy's Hospital for two years, matron of West Norfolk Hospital, Lynn, for two and a-half years, and sister at St. Marylebone Infirmary for two years. Miss Ashbee did district work for one year at Sydenham; at Springfield, Grimsby, she had two district nurses under her, and was matron of the Nurses' Institution there. We wish Miss Ashbee success in her present work.

Trimmer's Cottage Hospital, Farnham, Surrey.—Miss Martha Greening has been appointed Matron of this hospital. She was trained at the Evelina Hospital, Southwark Bridge Road, where she remained for nine years. Miss Greening was head nurse of male surgical wards at Guest Hospital, Dudley, for three years, and Sister of male accident and surgical division at the General Hospital, Nottingham, for the last six years. We congratulate Miss Greening on her appointment, and wish her success in her new work.

Royal Infirmary, Newcastle-on-Tyne.—Miss Emily Aston has been appointed matron and superintendent of nurses at this infirmary. She was trained in the Nightingale School of St. Thomas's Hospital, and had charge of a surgical ward and afterwards of St. Thomas's Home for Paying Patients. Miss Aston was lady superintendent of the General Hospital at Colombo for a year and ten months; took matron's duties for a short time at St. Mary Abbots Infirmary; was for three years assistant matron at the Royal Infirmary, Liverpool; for nearly two years matron of the Colonial Hospital, Gibraltar; and for eighteen months matron of the Strangers' Hospital, Rio de Janeiro. Miss Aston holds excellent testimonials, and many good wishes accompany her to her new work.

### Royal British Nurses' Association.

A meeting to promote the interests of this association was held at Brighton on Thursday, November 7th, at the Sussex County Hospital, under the management of Miss Scott, lady superintendent. A deputation from London attended the meeting, consisting of the three honorary officers (Mr. Langton, Mr. Fardon, and Mrs. Dacre Craven), Dr. S. Coupland, and the secretary. Miss de Pledge was prevented by illness from fulfilling her engagement to be present.

### Presentation.

On leaving Nottingham General Hospital Miss Greening, whose appointment is noted in another column, was presented with an afternoon tea set by her patients, with a silver teapot by the nursing staff, and a case of silver spoons by the resident medical staff.

### Novelties for Nurses.

### HARROD'S STORES.

A visit to Harrod's stores can never be other than time well spent, and now that a department for nurses' requisites has been opened our readers, like the rest of the public, can be accommodated in the most complete and reasonable manner. The choice is a varied one and extends over a large range of articles. Caps are, perhaps, the first object to attract the eye, and very pretty and becoming some of them are. The popular "Sister Dora" is made up in two or three slight variations as regards style and material, an important detail being the tape at the back which draws the cap into shape and unties for washing. The sister "Mary" is graceful and is a shape that will be much appreciated for its simplicity. It is finished off with ends that hang half-way down the back, which are occasionally preferred to strings under the chin. Quite one of the most charming of these confections, however, is one appropriately styled "The Matron." Hitherto matrons have been rather left out in the cold with regard to headgear, and while nurses have been abundantly catered for the more elaborate style usually adopted by the head of the nursing department has been a question of individual design or manufacture. The little cap we mention, which has only to be seen to be admired, is made of fine muslin trimmed with Valenciennes lace, three or four tiny frills in front making a becoming framework to the face. Frilled strings tie in a soft bow of muslin lace under the chin. It seems especially designed to supply what has been distinctly felt to be a want. There are several qualities in linen aprons, fine hem-stitched ones being a special line. Lawn sleeves and plain tucked cambric bonnet strings are likewise found in this department, the cost in all cases being small and the materials used being exceptionally good. Dress materials are another strong point at this excellent emporium. Prints, cambrics, linens, zephyrs, in every conceivable shade and pattern, afford a wide choice to the purchaser. A butcher-blue linen, quite fast in colour, would make an ideal "sister's" dress. It is soft, and will lend itself readily to draping, though substantial enough to withstand any amount of wear and tear. Especially worthy also of notice are the checked, striped, and plain fabrics, all of which are guaranteed to stand the ordeal of the wash-tub, and are pretty enough to suit the most exacting taste. Nurses in want of uniform will find their difficulties easily and cheaply solved at Harrod's Stores.

### THE ORNHO CORSELET BELT.

The "Ornholine" Manufacturing Company, of Newton Street Mills, Manchester, have produced a very excellent substitute for the corset in their Ornho belt. District nurses are largely adopting bicycle and tricycle to secure rapid transit from place to place, and they in common with lay women must find the corset an impediment to the healthy use of the bicycle. No support at all from whalebone or steel causes an untidy appearance, and nurses would lose their character for neatness and trimness were they entirely to dispense with it. Such a belt as the Ornho overcomes the difficulty. Whilst allowing full play for the lungs, it secures symmetry and compactness at the waist. The materials used are of the most durable description. A semi-transparent webbing forms the foundation, this material allowing a wholesome ventilation. The belt is bound with leather, with which the bones also are covered. The whole appearance is very neat, and the belt generally will, we think, find favour with all ladies who cycle or desire to discard the ordinary corset.

### The Book World for Women and Nurses.

[We invite Correspondence, Criticism, Enquiries, and Notes on Books likely to interest Women and Nurses. Address, Editor, The Hospital (Nurses' Book World), 428, Strand, W.C.]

### MAGAZINES OF THE MONTH.

### The Westminster Review.

Whilst the *Westminster Review* maintains a uniform excellence this month, there are one or two articles which are of more than ordinary interest. Mr. Burton's chapter on "An Appreciation of Russian Fictional Literature," for instance, is brightly and thoughtfully written, as is also a "Gallery of Australasian Singers," by Oliphant Smeaton, which, though dealing in a highly appreciative manner with the works of certain specialised poets, is more concerned in explaining the absence of a "past" in Australian literature—a literature which is notable for promise, as the writer remarks rather than for fruition. On a subject of greater topical moment is Mrs. Ritchie's "A National Waste." "The question before us now," to quote the writer's words, "is a waste of national force, which, if better directed, might produce more happiness, better morals, and a greater chance of national survival. The national force that is being wasted is woman." Mrs. Ritchie works out her article in a genuinely reflective and masterly manner.

### The Humanitarian.

Dr. Schofield's "Cycling for Ladies" is a carefully-considered article in the November number of this magazine. This contains many salutary suggestions, which ought to prove of value to the vast army of female bicyclists daily on the increase amongst us. "The Pharmacy of the Soul" is continued by the editor, and sustains the interest it created in the earlier chapters.

### The Cornhill Magazine.

A particularly bright account of "Early Advertisements" we find in these pages, giving a very good general view, too, of the whole subject. This is an unsigned article, as are four others in the present issue. Mr. Crockett's "Cleg Kelly" has reached its thirty-seventh chapter, and "The Sowers" its fortieth.

### The Leisure Hour.

There is always something of educative value in the contents of the *Leisure Hour*, and the present issue proves to be no exception to the rule. Mr. John Dennis' "Talk About Talk" is full of suggestive remarks. We wish it were longer. "Thomas Carlyle," Chapter I., by Mrs. Mayo, is commenced in this number.

In Blackwood's Magazine for this month there is an article on a remarkable village just above the valley of the Rhone, called Leysin-sur-Aigle; the writer designates it "The Village of Perfect Health." Certainly it reads like a fairy story, and though English people, so far, are not very well acquainted with the locality, yet to Russians, Italians, and other foreigners it is already known on account of its wonderful climate, immunity from wind, and abundance of sunshine.

The Oxford University Press has just supplied a want long felt in the shape of a neat and clearly printed guide to English ancient and modern hymns. This little volume is called "Oxford Helps to the Use of Hymns Ancient and Modern," and is published at the modest sum of 6d. The work serves to show what hymns are appropriate for use on every occasion daily throughout the year, on the feasts and fasts of the Church, in a convenient compass.

### A Coincidence.

It may interest our readers to have their attention drawn to the fact that the following notices appeared the same day in the *Times*:—

In Memoriam.

In most loving remembrance of my beloved husband, SIR ANDREW CLARK, Bart., who entered into his rest Nov. 6th, 1893.

At last shall break eternal dawn,
At last shall breathe eternal air
And Life be love, and Death be dead.
May we be there—we may be there!

In loving memory of DR. JOHN CHARLES STEELE, of Guy's Hospital, who died Nov. 6th, 1892.

## Figures and Tables

**Fig 10 f1:**
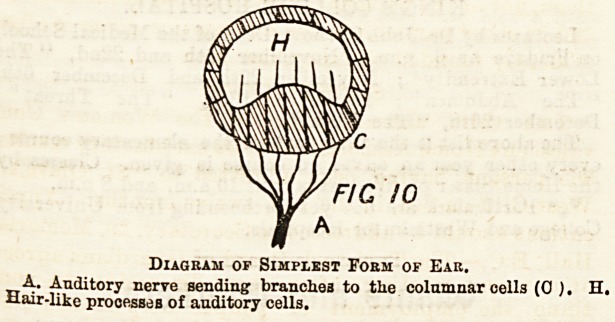


**Fig 11 f2:**